# In Memoriam to Vittorio Ricci, Professor of Pathology at Pavia University

**DOI:** 10.3390/toxins12060401

**Published:** 2020-06-17

**Authors:** Patrice Boquet

**Affiliations:** Department of Clinical Bacteriology, Nice University Hospital, 151 Route St Antoine de Ginestiere, 06202 Nice, France; patrice.boquet@free.fr

My great friend Vittorio Ricci died suddenly on 4 May 2020.

I met Vittorio for the first time in Washington in 1997, where the big meeting on gastroenterology was taking place. It was at one of the poster sessions, where Vittorio was presenting data on the vacuolating toxin of *Helicobacter pylori* (VacA), that we actively spoke for the first time. The study of this toxin was one of my laboratory’s new topics in Nice. One of my students, Antoine Galmiche, had started his PhD on the mode of action of VacA, which was unknown at that time (except for its vacuolating activity), and as a mise en jambes, he was trying to reproduce data on VacA from a prominent Italian group working on VacA. However, Antoine could not at all reproduce these data and complained to me that the topic was quite cumbersome. I discussed this problem with Vittorio and we imagined some putative mode of action for this toxin, which is fascinating for a cell biologist.

At the beginning of 1999, I got a letter from Vittorio asking for a post-doctoral year in my laboratory in Nice in order to study VacA. Vittorio easily obtained financial support from INSERM. In the fall of 1999, Vittorio came to Nice, and, using the cell biology tools that we had, we started to study the mechanism by which VacA is taken up by cells. Rapidly, he found that VacA was endocytosed by a clathrin-independent pathway and that lipid rafts were absolutely required for toxin cell binding [1]. Later on, he helped Antoine with his thesis, who finally found that mitochondria were targets for VacA and induced cell death [2]. All these data were fully confirmed by different groups around the world.

Vittorio returned back to Italy in 2000 but our fruitful collaboration was still ongoing between Pavia and Nice. In Nice, a new student, Nils Gauthier, started his PhD on the study of the intracellular trafficking of VacA. With the great help of Vittorio, who furnished a fully active fluorescent labeled VacA, Nils beautifully demonstrated with this tool that VacA was endocytosed by a newly described clathrin-independent pathway named the CLIC/GEEC mechanism [3]. In fact, this is actually a key point to understanding why T and B lymphocytes are sensitive to and killed by VacA.

Again, some years later, in closed collaboration with Vittorio, we showed that the CagA molecule (cytotoxin associated with VacA), injected by a Type VI mechanism by the *Helicobacter pylori* into gastric cells (when the bacterium colonizes the gastric epithelium) [4], in fact counteracted the deleterious effect of VacA on the gastric epithelium, thus deciphering a new virulence bacterial strategy [5,6].

Just before the disappearance of Vittorio, and in collaboration with my ex-student Nils Gauthiers, who actually works at the IFOM in Milan, I was actively working on new mechanisms of CagA and VacA. In memory of Vittorio, we hope to complete these works, which will be dedicated to Vittorio.

Goodbye, Vittorio. You were like King Midas; everything you touched became gold.
